# A novel prognostic prediction model based on seven immune-related RNAs for predicting overall survival of patients in early cervical squamous cell carcinoma

**DOI:** 10.1186/s12920-021-00885-3

**Published:** 2021-02-15

**Authors:** Rui Qin, Lu Cao, Cong Ye, Junrong Wang, Ziqian Sun

**Affiliations:** Department of Obstetrics and Gynecology, The Third Hospital of Jilin University, No 126, Xiantai Street, Changchun, Jilin 130033 People’s Republic of China

**Keywords:** Early cervical squamous cell carcinoma, Immune-related RNAs signature, Prognostic prediction model

## Abstract

**Background:**

In this study, we aimed to mine immune-related RNAs expressed in early cervical squamous cell carcinoma to construct prognostic prediction models.

**Methods:**

The RNA sequencing data of 309 cervical squamous cell carcinoma (CSCC) cases, including data of individuals with available clinical information, were obtained from The Cancer Genome Atlas (TCGA) database. We included 181 early-stage CSCC tumor samples with clinical survival and prognosis information (training dataset). Then, we downloaded the GSE44001 gene expression profile data from the National Center for Biotechnology Information Gene Expression Omnibus (validation dataset). Gene ontology annotation and the Kyoto Encyclopedia of Genes and Genomes pathway analyses were used to analyze the biological functions of differentially expressed immune-related genes (DEIRGs). We established protein–protein interactions and competing endogenous RNA networks using Cytoscape. Using the Kaplan–Meier method, we evaluated the association between the high- and low-risk groups and the actual survival and prognosis information. Our univariate and multivariate Cox regression analyses screened for independent prognostic factors.

**Results:**

We identified seven prognosis-related signature genes (*RBAKDN*, *CXCL2*, *ZAP70*, *CLEC2D*, *CD27*, *KLRB1*, *VCAM1*), the expression of which was markedly associated with overall survival (OS) in CSCC patients. Also, the risk score of the seven-gene signature discripted superior ability to categorize CSCC patients into high-risk and low-risk groups, with a observablydifferent OS in the training and validation datasets. We screened two independent prognostic factors (Pathologic N and prognostic score model status) that correlated significantly by univariate and multivariate Cox regression analyses in the TCGA dataset. To further explore the potential mechanism of immune-related genes, we observed associated essential high-risk genes with a cytokine–cytokine receptor interaction.

**Conclusions:**

This study established an immune-related RNA signature, which provided a reliable prognostic tool and may be of great significance for determining immune-related biomarkers in CSCC.

## Background

Cervical cancer (CC) is the second primary cause of death for women worldwide, accounting for more than 260,000 deaths each year (1). Cervical squamous cell carcinoma (CSCC) is the most common type of CC (2). Cervical intraepithelial neoplasia (CIN) is a precancerous lesion that is strongly related to CC and includes CIN I–III, each of which reflects the successive progression of CC (3). The early clinical symptoms of CC are often undetectable. Therefore, it is crucial to find markers of early-stage CC to improve the prevention and treatment of this disease. Biomarker discovery is a key to the early diagnosis of CC and improvements in cure and survival rates.

Recently, immunotherapy has been proved to be a vigorous modality to treat multifarious conditions, given that our understanding of immune system function has improved in essence (4). As a Human Papillomavirus (HPV)-driven cancer, CC appears to be at least partly mediated by the immune system. Checkpoint immunotherapy has shown significant efficacy in lung, bladder, renal, and head and neck cancers (5). CC will hopefully, at some point, be considered a tumor that benefits from immunotherapeutic agents. Zhao et al. (6) found that MMP1 may be a novel biomarker for immunotherapy and prognostic assessment of patients with CC. Karpathiou et al. (7) indicated that PD-L1 and CTLA-4 immune cell expression was associated with lymph node metastasis and are, therefore, potential therapeutic targets in CC. Wang et al. (8) found that immune system-related genes referred to the T cell receptor (TCR) signaling pathway are associated with the overall survival (OS) of CSCC patients. Previous studies reported that immune checkpoints are initiated by ligand-receptor interactions that are simply blocked by antibodies or modulated by recombinant forms of ligands or receptors. Thus, these immune checkpoints are attractive drug targets for cancer therapeutics (9). Furthermore, Yu et al. (10) analyzed the gene expression data from The Cancer Genome Atlas (TCGA) and constructed a risk model based on 26 DElncRNAs. The results indicated that the risk prediction model had a properly high accuracy to predict the prognosis of CSCC patients, suggesting that these DElncRNAs were possibly related to CSCC prognosis. Zhou et al. (11) established a regression model by CSCC gene expression, the prediction accuracy of which for CSCC was high. Although previous studies have identified a number of gene markers in the occurrence and recurrence of CSCC, further research is needed on the impact of gene characteristics on OS survival and prognosis.

In our study, we first analyzed the transcriptome to determine the expression level characteristics of early-stage CSCC cases in TCGA database. After assessing immune-related genes, we screened the RNAs closely related to CSCC and the immune system. The results allowed us to construct a prognostic model based on prognosis-related RNAs.

## Methods

### Data collection and preprocessing

As of March 12, 2020, the RNA sequencing data of 309 patients with CSCC, including individuals whose clinical information was available, were downloaded from TCGA. After analyzing the corresponding clinical data, only the early-stage CSCC tumor samples, marked as “stage I” and “stage II” in the “Pathologic stage” category, were retained. Finally, we included 181 early-stage CSCC tumor samples with clinical survival-related prognostic information. These cases served as the training dataset to construct a prognostic model. Therefore, we downloaded the GSE44001 gene expression profile data from the National Center for Biotechnology Information Gene Expression Omnibus (http://www.ncbi.nlm.nih.gov/geo/) (17) based on the platform of the GPL14951 (18) Illumina HumanHT-12 WG-DASL V4.0 R2 Expression BeadChip. The GSE44001 dataset included 300 CC samples with survival-related prognostic information; these cases served as the validation dataset to construct the prognostic model.

### Screening of differentially expressed RNAs and differentially expressed immune-regulated genes

We used 4528 lncRNAs and 19,194 protein-coding genes in the HUGO Gene Nomenclature Committee (http://www.genenames.org/) (20) to identify the lncRNAs and mRNAs in the expression profile. Then, we divided the cancer cases into the poor prognostic group (A group: cases with a survival time less than three years and death) or the good prognostic group (B group: cases with a survival time greater than five years) (21). Next, limma package (Version 3.34.7, https://bioconductor.org/packages/release/bioc/html/limma.html) (22) was used to screen the differentially expressed RNAs (DERs) in the poor prognostic and good prognostic groups using the false discovery rate (FDR) threshold of < 0.05 and |log2FC|> 1 (2 times). According to the expression value of the DERs in the training dataset, the heatmap package in R 3.4.1 (Version 1.0.8; https://cran.r-project.org/web/packages/pheatmap/index.html) (23) was used to perform bidirectional hierarchical clustering on the expression of DER values based on the centered Pearson correlation algorithm (24).

Moreover, in the AmiGO 2 (http://amigo.geneontology.org/amigo) database, “immune” was used as the keyword to search the biological processes related to immunity, after which we downloaded the genes involved in immune-related biological processes. We also downloaded all the related pathways and genes involved in the “immune” entry from the KEGG database. We obtained differentially expressed immune-regulated genes (DEIRGs) by crossing the previously acquired DEGs list with the list of immune-related genes.

### Construction of the co-expression network

We used the cor.test function in R3.4.1 (https://stat.ethz.ch/R-manual/R-devel/library/stats/html/cor.test.html) to calculate the Pearson correlation coefficient (PCC) (25) between the expression level of the intersecting DEGs in the CSCC training dataset and the DElncRNAs, which was performed to construct a co-expression network of DElncRNA and intersecting DEGs. This network was visualized using Cytoscape software (version 3.6.1, https://cytoscape.org/) (26). An analysis of the Gene Ontology (GO) (22) biological process, and Kyoto Encyclopedia of Genes and Genomes (KEGG) pathway enrichment annotation, based on the Database for Annotation, Visualization and Integrated Discovery (27) (version 6.8, https://david.ncifcrf.gov/) (28, 29), was performed on the intersecting DEGs with P < 0.05 as the threshold.

### Construction of a prognostic model based on DERs

Our univariate and multivariate Cox regression analyses, using the survival package in R3.4.4 (version 2.41-1, http://bioconductor.org/packages/survivalr) (30) were used to screen the DERs that were significantly related to overall survival (OS) and prognoses based on the CSCC tumor samples in the training dataset; a P < 0.05 was used as the threshold according to the log-rank test. A LASSO Cox regression (31) model, based on the L1-penalized regularization regression algorithm in the penalized package (Version 0.9-50; http://bioconductor.org/packages/penalized/) (32) in R3.4.1, was used to screen out the optimized combinations of the prognosis-related signature DERs (the optimized parameter “lambda” in the model was selected and calculated through a 1000 times cross validation likelihood cycle). The Kaplan–Meier (KM) (33) survival curve in the R3.4.1 language survival package (version 2.41-1) (30) was used to evaluate the association between patients OS time and the expression of the optimized DERs. Then, based on the prognostic coefficient of genes in the optimal DER combinations, obtained from the previous regression algorithm, we constructed a risk prediction model, based on the gene expression level in the training dataset, and calculated the risk score of each sample. The prognostic score (PS) was calculated as follows:

$${\text{PS}} = \sum\upbeta _{{{\text{DER}}}} {\text{s }} \times {\text{Exp}}_{{{\text{DERs}}}}$$

where βDERs represents the prognostic coefficient of the optimized DERs in the LASSO algorithm, andExp_DERs_ represents the expression level of the corresponding DERs in the training dataset.

With the median PS as the cutoff, the samples in the training dataset were separate into high-risk and low-risk groups, and the correlation between the risk model and prognosis was evaluated by a KM (33) survival curve in the R3.4.1 language survival package (version 2.41-1) (30). Moreover, we extracted the expression value of the target DEGs from the GSE44001 validation dataset. Each sample’s PS score was obtained according to the equation described above by using the β value from training dataset. The validation dataset samples were divided into the high- and low-risk sample groups based on the PS value of the validation dataset. The survival package (version 2.41-1) and the KM curve method in R3.4.1 (30) was used to evaluate the association between the high- and low-risk groups and the actual survival-related prognosis information for the validation dataset samples.

### Screening of independent prognostic clinical factors

We performed the univariate and multivariate Cox regression analyses using the R3.4.1 language survival package (Version 2.41-1) (34) and screened the independent prognostic clinical factors in CSCC samples from the TCGA dataset with a threshold of P < 0.05 from the log-rank test to screen for significant correlations.

To explore the association between the independent prognostic clinical factors and the risk groups, we performed a clinical factor stratification analysis of the selected independent prognostic factors that were significantly correlated: the samples were divided into different groups according to the clinical factors, and a correlation analysis of the risk prognostic models was performed for these different groups.

According to the PS values, the samples in the TCGA training dataset were separatedinto high-risk and low-risk groups. Then, the limma package was used to analyze the differences between the mRNA expression matrices of the samples in the high- and low-risk groups. Similarly, an FDR < 0.05 and |logFC|> 0.5 were used as thresholds for determining significant differences. Then, an analysis of the GO (22), BP, and KEGG pathway enrichment annotation based on The DAVID (version 6.8, https://david.ncifcrf.gov/) (28, 29) was performed on the intersecting DEGs with P < 0.05 as the threshold.

## Results

### Screening of significant DEGs

**Fig. 1:**
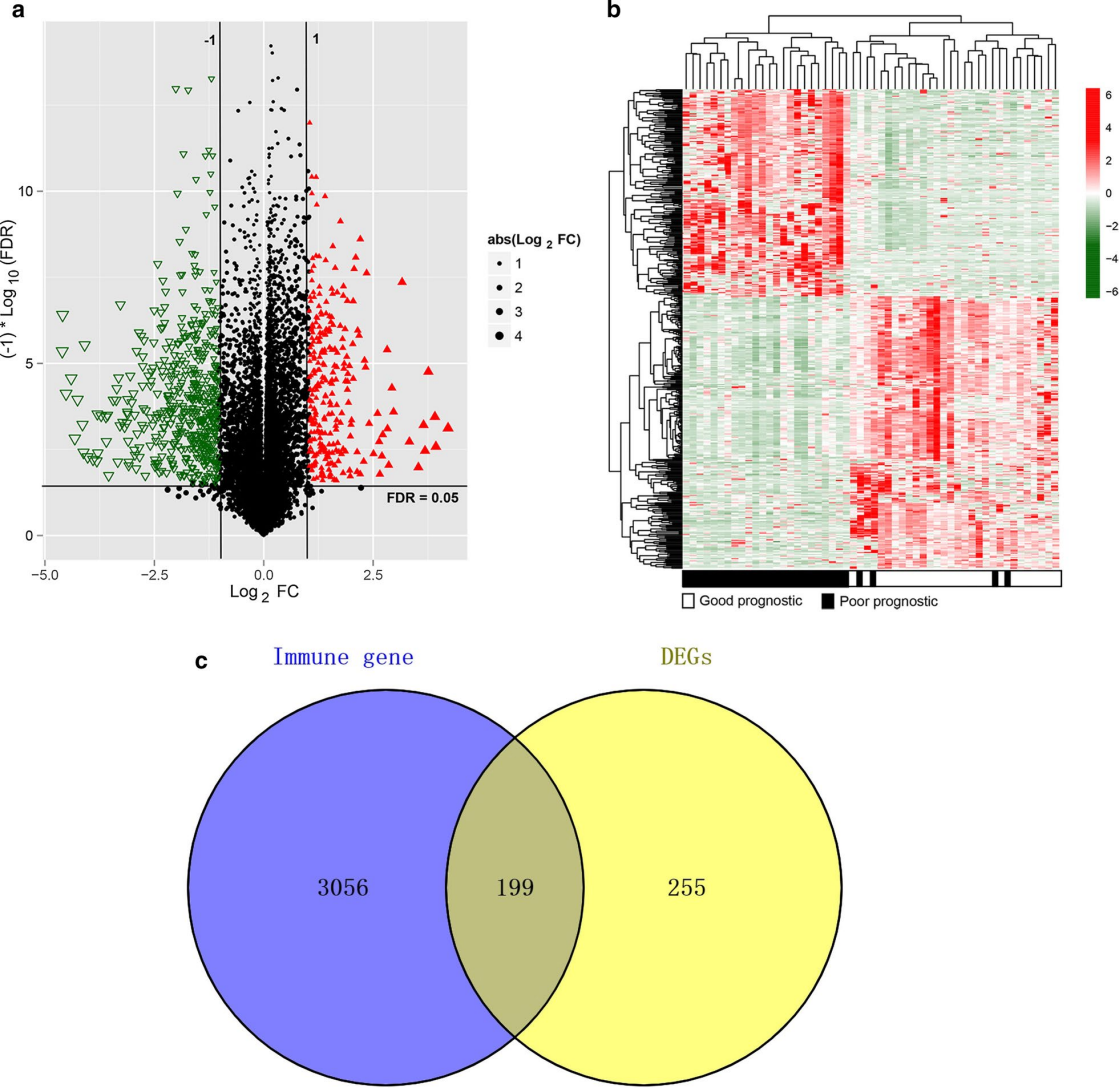
Screening of DEGs. **a** Effect size (log_2_FC)–log_10_ (FDR), shown by a volcano plot. The red triangle and green del triangle represent the significantly upregulated and down-regulated DERs, respectively. The horizontal dotted line represents FDR < 0.05, the two vertical dotted lines represent |log_2_FC|> 1, and the size of the point represents the absolute value of log_2_FC. The larger the value, the larger the point; **b** Bidirectional hierarchical clustering heatmap based on the expression level of DERs. **c** Venn diagram shows the comparison between immune-related genes and DEGs with DERs in the dataset. DEGs, differentially expressed genes; DERs, differentially expressed RNAs; FDR, false discovery rate

With intersections among the TCGA, and GSE44001 datasets, 343 lncRNAs and 15,735 mRNAs were obtained. Then, we divided the samples into the poor prognostic and good prognostic groups, which included 26 and 28 samples, respectively. In all, 454 DERs (197 down-regulated DERs and 257 upregulated DERs) were screened using the limma package (Fig. 1a). The heatmap showed that the samples were clustered in two different directions (Fig. 1b).

Moreover, 3020 unique genes related to immune GO, 817 unique genes related to immune KEGG, 582 intersecting genes, and 3255 union genes were obtained from the database. When the immune-related genes were compared with the DEG dataset, 199 shared genes were obtained (Fig. 1c) and were used for the next analysis.

### Construction of the co-expression network

**Fig. 2:**
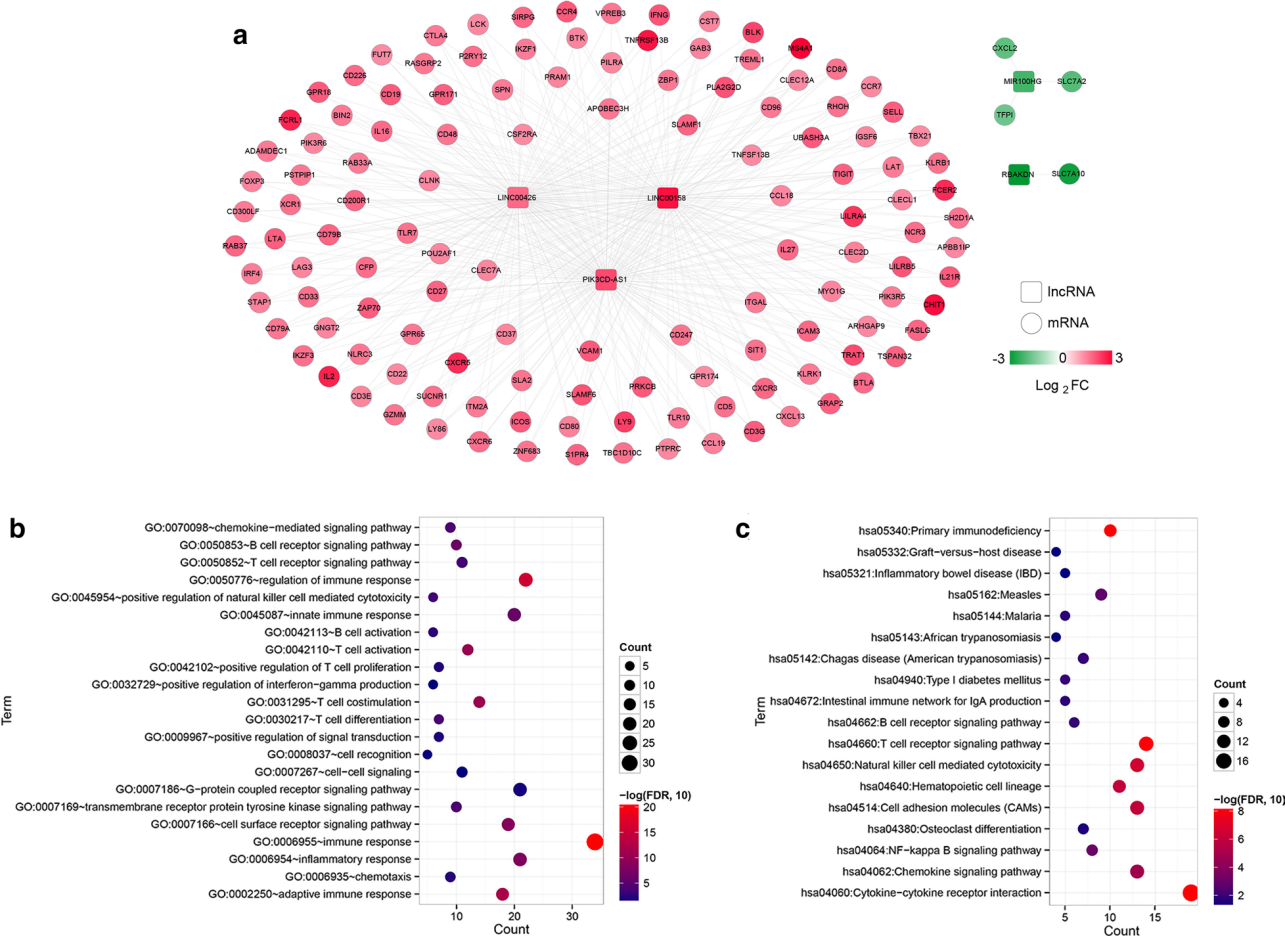
Construction of the co-expression network. **a** There were significant differences in the expressions of lncRNAs and intersecting mRNAs in the co-expression network. The squares and circles represent lncRNA and mRNA, respectively, and the change in color from green to red represents a significant down-regulation to upregulation of logFC expression. In the co-expression network, the biological process (**b**) and KEGG signal pathway (**c**) in the scatter plot are related to gene enrichment; the horizontal axis represents the gene number, the vertical axis shows the item name, and the color and size of the point represent a significant FDR value. The closer the color of the point is to red, the greater the color and the greater the significance. KEGG, Kyoto Encyclopedia of Genes and Genomes

The PCC was calculated between the expression level of a gene in the CSCC tumor samples from the TCGA dataset and that of the intersecting DEGs and DElncRNAs. In all, 375 pairs of connections were obtained with a cutoff expression correlation coefficient higher than 0.4, which was used to construct the DElncRNA and DEG intersection co-expression network. As shown in Figs. 2, 5 lncRNAs and 130 mRNAs were included in this network. Interestingly, we observed antisense lncRNAs that were co-expressed with sense mRNAs, such as the RBAKDN-SLC7A10, LINC00158-MS4A1/TNFRSF13B-LINC00426, and LINC00158-FCRL1-PIK3CD-AS1 pairs. Moreover, we performed GO and KEGG analyses for the DEGs; overall, 22 BPs and 18 KEGG pathways were screened. The GO analysis indicated that the DEGs were primarily involved in immune response (34 genes, such as *CXCL2*, *ZAP70*, *CD27*), regulation of immune response (22 genes, such as *VCAM1*, *KLRB1*, *CLEC2D*), inflammatory response (21 genes, such as *CXCL2*, *ZAP70*, *CD27*), and the innate immune response (20 genes, such as *ZAP70*, *CLEC7A*). The KEGG pathway analysis revealed that the DEGs were primarily enriched in pathways related to cytokine − cytokine receptor interaction (19 genes, such as *CXCL2*, *TNFRSF13*, *CD27*), TCR signaling pathway (14 genes, such as *ZAP70*, *CD8A*), and Primary immunodeficiency (10 genes, such as ZAP70, *TNFRSF13B*) (Fig. 2b).

### Constructing the prognostic prediction model

**Fig. 3:**
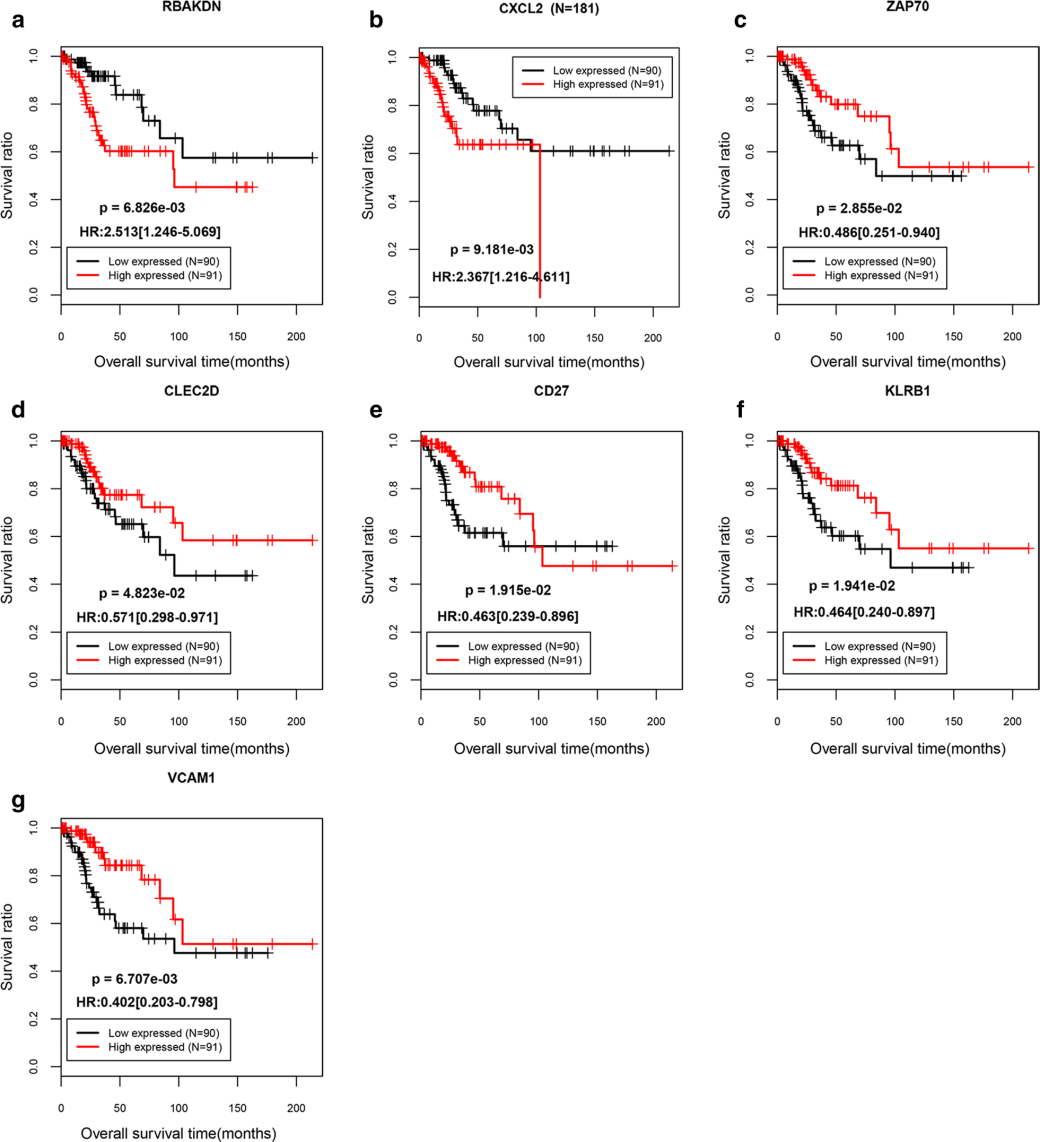
The KM curves of the 7 optimized DERs. A-G indicated the *RBAKDN*, *CXCL2*, *ZAP70*, *CLEC2D*, *CD27*, *KLRB1*, and *VCAM1*, respectively. KM, Kaplan–Meier; DERs, differentially expressed RNAs

In all, 123 DERs that were markedly associated with CC prognosis were obtained by univariate Cox regression analysis using the cutoff of the log-rank P < 0.05. We then obtained 31 independent DEGs that were dramatically related to prognosis by multivariate Cox regression analysis. Subsequently, seven optimized DER groups (*RBAKDN, CXCL2*, *ZAP70*, *CLEC2D*, *CD27*, *KLRB1*, *VCAM1*) were selected through Cox-Proportional hazard (Cox-PH) regression models on basis of the L1-penalized regularization regression algorithm in penalized the package of R. Among the seven genes, *RBAKDN* and *CXCL2* were risk factors (hazard ratio (HR) > 1), whereas *ZAP70*, *CLEC2D*, *CD27*, *KLRB1*, and *VCAM1* were identified as protective factors (HR < 1). Additional information on these seven genes is shown in Table 1. Moreover, the KM curves indicated that the low expression of *RBAKDN* and *CXCL2* was associated with good prognosis, while the high expression of *ZAP70*, *CLEC2D*, *CD27*, *KLRB1*, and *VCAM1* was related to better OS time than their high expression (Fig. 3).

**Table 1:** Optimize the signature DERs combination information table

Symbol	Type	Univariate Cox regression analysis			LASSO coefficient
		HR	95%CI	P value	
RBAKDN	lncRNA	11.950	4.529–31.54	5.421E−07	1.87328
CXCL2	mRNA	1.427	1.192–1.709	1.098E−04	0.35901
ZAP70	mRNA	0.493	0.323–0.754	1.107E−03	− 0.30060
CLEC2D	mRNA	0.329	0.167–0.651	1.399E−03	− 0.47174
CD27	mRNA	0.595	0.428–0.827	2.006E−03	− 0.06947
KLRB1	mRNA	0.498	0.318–0.781	2.403E−03	− 0.03436
VCAM1	mRNA	0.542	0.363–0.809	2.768E−03	− 0.14466

HR, hazard ratio; CI, confidence interval

### Evaluation and comparison of the effectiveness of the prognostic risk prediction model

**Fig. 4:**
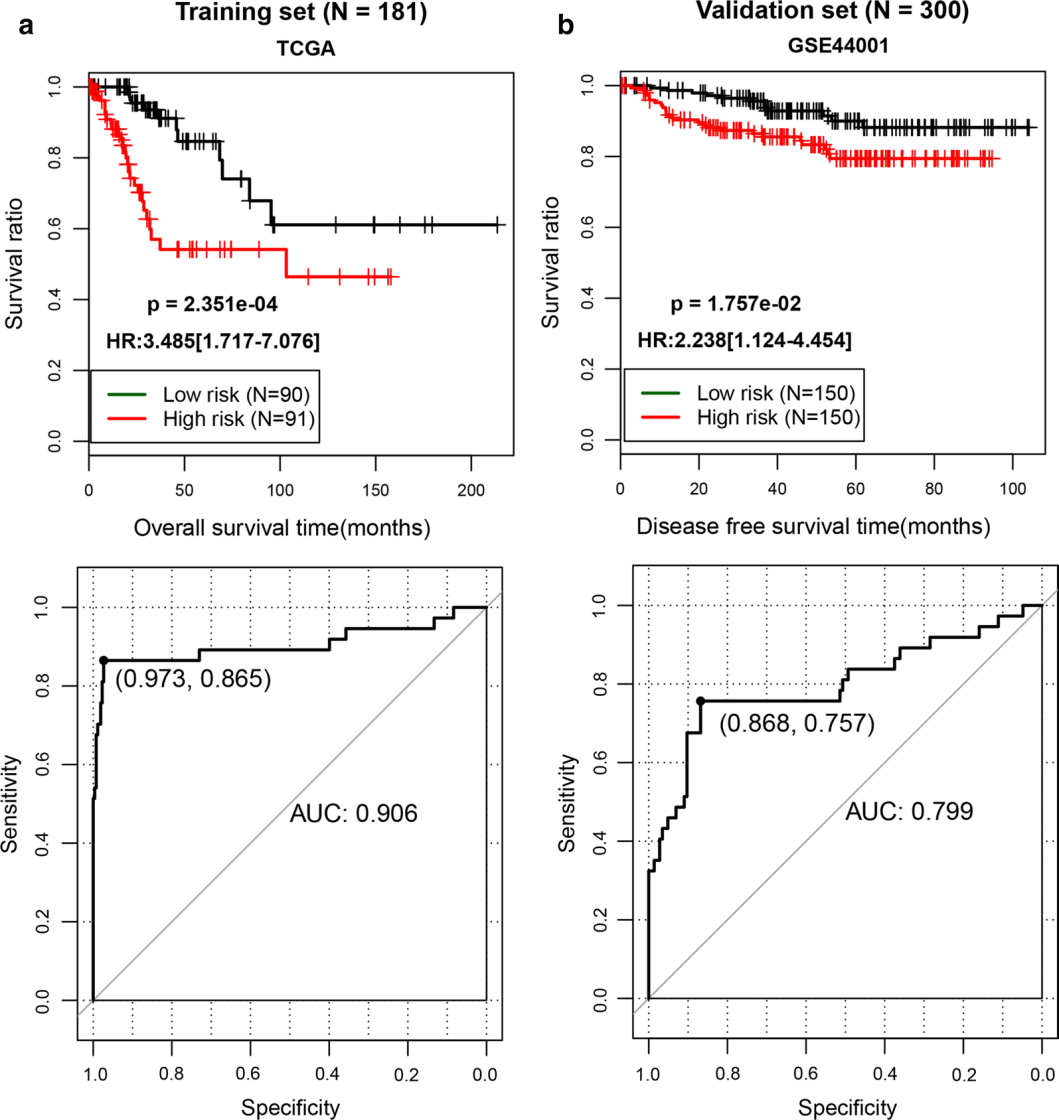
Upper panel: TCGA (**a**) and GSE44001 (**b**) samples are based on the KM curve of the PS prediction model and the prognosis; the green and red curves represent low- and high-risk samples, respectively. Lower panel: ROC curve of the prediction results based on the PS prognostic model. The numbers in brackets represent the specificity and sensitivity corresponding to the ROC curve. TCGA, The Cancer Genome Atlas; KM, Kaplan–Meier; PS, prognostic score; ROC, receiver operating characteristic

The PS model was constructed on basis of the seven optimum DERs, found by LASSO algorithm, and their expression level in the TCGA training dataset. Then, we divided the TCGA training dataset and the GSE44001 validation dataset into high-risk and low- risk groups. The TCGA dataset’s KM curves were used to evaluate the connection between the high- and low-risk groups and actual prognostic information for CC. As shown in Fig. 4, we found that samples from low-risk cases in the TCGA dataset had a better survival prognosis (P = 2.351e−04, HR = 3.485[1.717–7.076], AUC = 0.906); there was a similar trend for the GSE44001 validation dataset (P = 1.57e-02, HR = 2.238[1.124–4.454], AUC = 0.799). The results revealed a notable correlation between the actual prognosis and the different risk groups obtained after the samples from the TCGA dataset and GSE44001 dataset were divided based on the PS model prediction.

### Screening and stratification of independent prognostic clinical factors

**Fig. 5:**
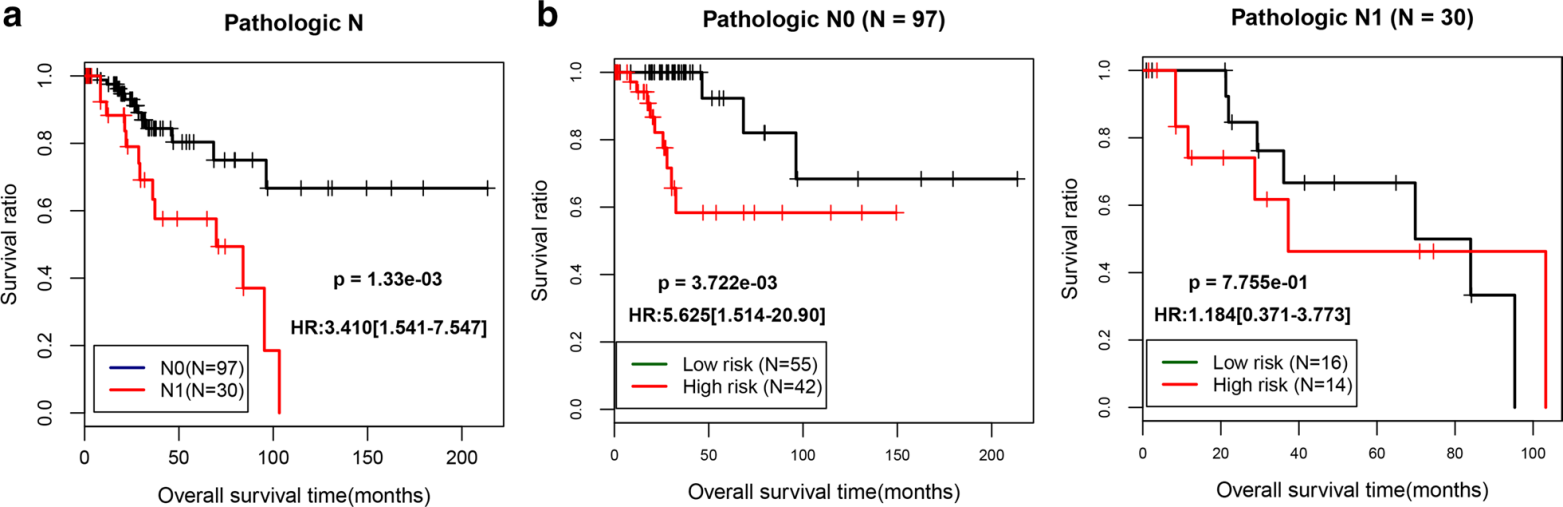
Screening and stratification of independent prognostic clinical factors. (**a**) The KM curve of the prognostic correlation of the Pathologic N stage is shown in the TCGA samples, and the black and red curves show the samples that were Pathologic N0 and N1 stages, respectively. (**b**) The samples in the demographic Pathologic N0 group are based on the KM curve of the PS prediction model and the prognosis; the black and red curves represent the low- and high-risk samples, respectively. KM, Kaplan–Meier; TCGA, The Cancer Genome Atlas; PS prognostic score

Two were significantly correlated independent prognostic factors (Pathologic N and PS model status) were screened by univariate and multivariate Cox regression analyses in the TCGA dataset (Table 2). We found that the lower the Pathologic N, the better the prognosis with respect to normal platelet indicators in CSCC tumor patients, which is consistent with the actual situation. We then separated the training dataset samples into the N0 and N1 sample groups, according to the Pathologic N status, and then analyzed the correlation between the prediction results of the PS prognostic model and the actual prognosis in each stratified sample dataset (Fig. 5). The KM curves of the Pathologic N status revealed that the samples in the N0 group had a better OS (P = 1.33e−03). Moreover, the KM curves of the Pathologic N0 and Pathologic N1 groups showed that the low-risk group had a better OS (P = 3.772e−03; P = 7.755e−01).

**Table 2:** Clinical factor screening information sheet

Clinical characteristics	TCGA (N = 181)	Uni-variables cox			Multi-variables cox		
		HR	95%CI	P	HR	95%CI	P
Age (years, mean ± sd)	47.06 ± 13.83	1.005	0.981–1.029	6.93E−01	–	–	–
Neoplasm histologic grade (G1/G2/G3/–)	11/77/77/16	1.219	0.655–2.269	5.30E−01	–	–	–
Pathologic M (M0/M1/–)	79/3/99	11.060	1.130- 108.2	9.48E−03	5.960	0.598–59.39	1.28E−01
Pathologic N (N0/N1/–)	97/30/54	3.410	1.541–7.547	1.33E−03	3.238	1.468–7.142	3.61E−03
Pathologic T (T1/T2/–)	99/50/32	1.263	0.584–2.729	5.52E−01	–	–	–
Pathologic stage (I/II)	123/58	0.820	0.396–1.696	5.87E−01	–	–	–
Number of pregnancies (0/1/2/3/over 3/–)	10/18/30/28/75/20	1.028	0.794–1.333	8.31E−01	–	–	–
Radio-therapy (yes/no/–)	105/53/23	1.190	0.571–2.479	6.42E−01	–	–	–
RS model status (high/low)	90/91	3.485	1.717–7.076	2.35E−04	2.518	1.122–5.651	2.51E−02
Vital status (dead/alive)	38/143	–	–	–	–	–	–
Overall survival time (months, mean ± sd)	37.83 ± 40.02	–	–	–	–	–	–

HR, hazard ratio; CI, confidence interval; N, number

### Functional analysis of high- and low-risk key genes

**Fig. 6:**
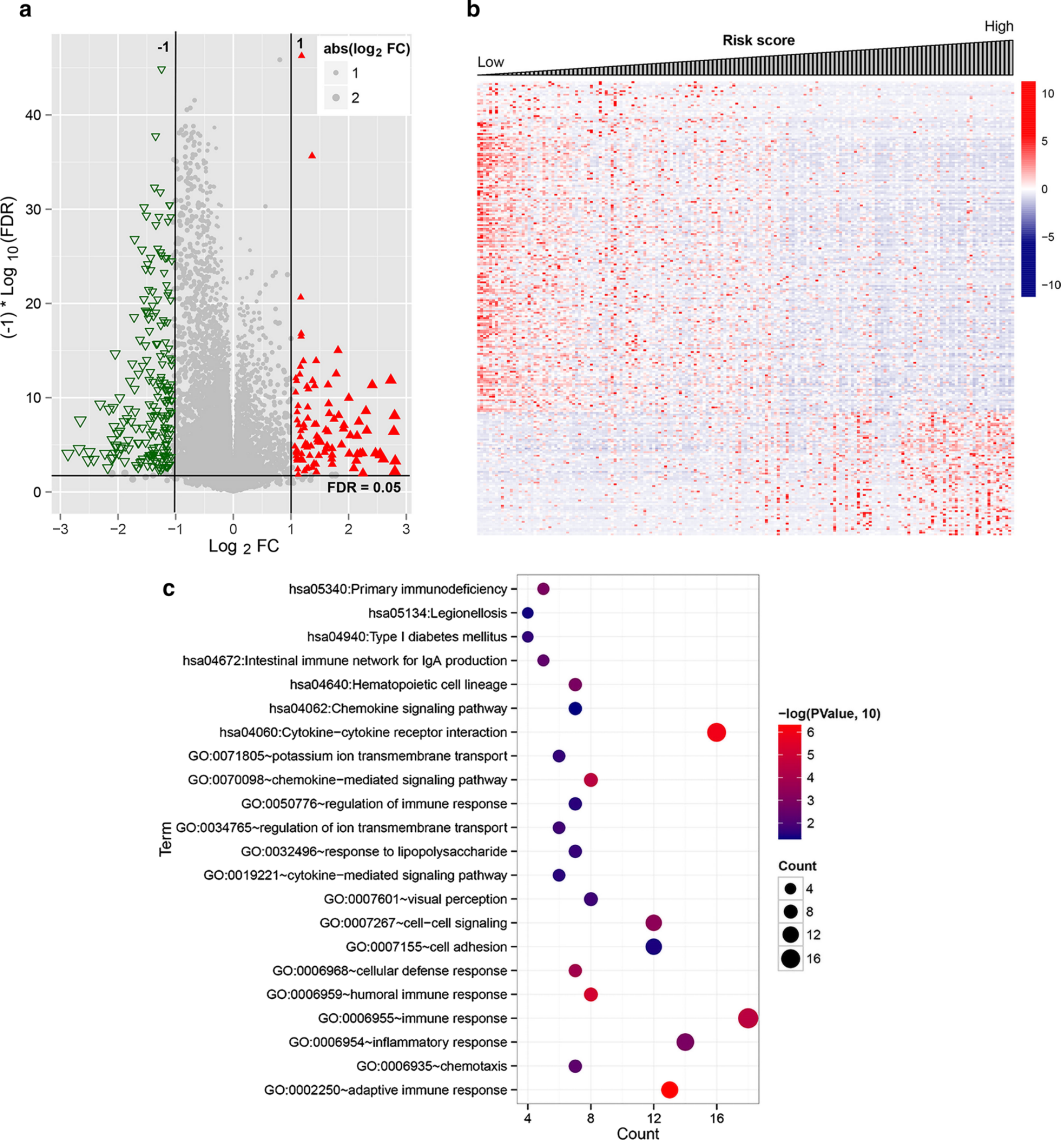
Functional analyses of high- and low-risk key genes. **a** In the effect size (log_2_FC)—log_1_0 (FDR) volcano map, the red triangle, and green triangle represent significantly upregulated and down-regulated DEGs, respectively, the horizontal dotted line represents FDR < 0.05, the two vertical dotted lines represent |log_2_FC|> 1, and the point size represents the absolute value of log_2_FC. The larger the value, the larger the point; **b** Based on the high- and low-risk groups, the changes in DEGs with the corresponding diagnostic scores from a low to a high expression level. **c** The horizontal axis represents the number of genes, the vertical axis represents the name of the item, and the color and size of the point represent the significant FDR value. The closer the color of the point is to red, the greater the color and the greater the significance. DEGs, differentially expressed genes; DERs, differentially expressed RNAs; FDR, false discovery rate

According to the PS value, the TCGA samples were separated into the high-risk and low-risk groups. In all, 254 DEGs (185 significantly down-regulated and 69 significantly upregulated genes) were screened using the limma package to analyze the differences between the expression matrices of the high- and low-risk group samples from the TCGA dataset (Fig. 6a). The heatmap showing the DEGs’ expression based on the risk score is shown in (Fig. 6b). The heatmap revealed that the DEGs’ expression level was significantly altered as the risk score changed increased. Then, a GO, BP, and KEGG pathways analysis was performed to determine the DEGs with a cutoff of P < 0.05. In all, 15 BPs and 7 KEGG pathways were screened. The GO analysis indicated that the DEGs were primarily involved in adaptive immune response (13 genes, such as *TNFRSF13B*, *THEMIS*) and immune response (18 genes, such as *CXCL2*) (Fig. 6c); the KEGG pathway analysis revealed that DEGs were primarily enriched in pathways related to cytokine–cytokine receptor interaction (*CXCL2*, *TNFRSF13B*) (Fig. 6c).

## Discussion

Although there has been substantial progress in the early diagnosis and treatment of CSCC, the incidence of this disease is still high, and it has a low diagnosis rate as well as a poor prognosis (35, 36). In our study, we used comprehensive bioinformatics analyses to identify seven prognosis-related signature genes (*RBAKDN, CXCL2*, *ZAP70*, *CLEC2D*, *CD27*, *KLRB1*, *VCAM1*). Their expression was significantly related to OS in CSCC patients. In addition, the risk score of the seven-gene feaure expressed its superior ability to categorize CSCC patients into high-risk and low-risk groups with markedly different OS in the training and validation datasets.

Additionally, to further distinguish the important genes that participate in the prognostic model, we constructed a protein–protein interaction (PPI) network. The results revealed that three hub genes were screened in the PPI network, *MS4A1*, *TNFRSF13B*, and *FCRL1*, which are significantly expressed. *TNFRSF13B*, a member of the TNF receptor superfamily, which occupied an important position in the proliferation and progression of cancer cell. Abo-Elfadl MT et al. (37) reported that *TNFRSF13B* silencing could be a therapeutic target for breast cancer subtype. Fc receptor-like 1 (*FCRL1*) is a novel B cell receptor (BCR) co-receptor. Zhao et al. (38) revealed a vital BCR signal enhancement role of *FCRL1* via its internal B cell immune synapse recruitment and subsequent c-Abl recruitment on BCR cross-linking. Furthermore, KEGG and GO analyses expressed that the genes were enriched in the immune response pathway. In particular, these results suggest the importance of genes associated with the immune response.

Moreover, among the seven prognosis-related signature genes, *RBAKDN* and *CXCL2* were both determined to be risk factors. A previous investigation found that the chemokine (C-X-C motif) ligand 2 (*CXCL2*) was originally characterized as a neutrophil chemokine. Specifically, *CXCL2* could improved the expansion of monocytic MDSCs (mo-MDSCs) a subtype of MDSCs. Shi et al. (39) indicated that *CXCL2* was expressed in tumor cells and tumor-infiltrating CD11b myeloid cells, which shows *CXCL2*′s novel role in increasing mo-MDSC generation by favoring the differentiation of bone marrow cells in tumor-bearing conditions. This suggests that inhibiting the levels of *CXCL1* and *CXCL2* could reduce mo-MDSC generation and promote host immunosurveillance. Zhang et al. (40) reported that A-kinase-interacting protein 1 is crucial in CC angiogenesis and growth because it functions to elevate the levels of the NF-κB-dependent chemokines *CXCL1*, *CXCL2*, and *CXCL8.* Zheng et al. (41) revealed that chemokine *CXCL2* induced lung cancer-associated transcript 1 (LUCAT1) overexpression and that the CXCL2β axis is a potential therapeutic target and molecular biomarker for clear cell renal cell carcinoma (ccRCC). All these results indicated that *CXCL2* could induce the production of adverse factors, which could contribute to CSCC’s poor prognosis. These results are consistent with ours and suggest that *CXCL2* is highly valuable for predicting the survival and prognosis of patients with CSCC. However, no studies have considered *RBAKDN’s* possible role in cancer.

To further explore the potential mechanism of immune-related genes, we observed that high-risk key genes were associated with the cytokine–cytokine receptor interaction pathway. The TRAIL/TRAIL-R system was regulated by Macrophages and neutrophils via cytokines to remove cancer cells (42). Li et al. (43) indicated the bistability of the network between cytokines and tumor immunity. This model has shown that tumors can take advantage of this bistability to improve immunosuppression. Eliminating this interaction means the immune system can return to an immune-boosting state. These results suggest that certain differentially expressed immune-related genes involved in cytokine–cytokine receptor interaction contribute to longer OS.

## Conclusions

In summary, we identified seven prognosis-related signature genes (*RBAKDN, CXCL2*, *ZAP70*, *CLEC2D*, *CD27*, *KLRB1*, *VCAM1*), the expression of which significantly correlated with OS in CSCC patients. Also, the risk score of the seven-gene signature demonstrated superior ability to divide CSCC patients into high-risk and low-risk groups, each of which had a markedly different OS in the training dataset and validation datasets. Two significantly correlated independent prognostic factors (Pathologic N and PS model status) were screened by univariate and multivariate Cox regression analyses in the TCGA dataset. To further explore the potential mechanism of immune-related genes, we observed that high-risk key genes were related to cytokine–cytokine receptor interaction.

## Data Availability

The datasets supporting the conclusions of this article are available in the [GSE44001] and [TCGA] repository, [https://www.ncbi.nlm.nih.gov/geo/query/acc.cgi?acc=GSE44001] and [https://xenabrowser.net/datapages/?cohort=GDC%20TCGA%20Cervical%20Cancer%20(CESC)&removeHub=https%3A%2F%2Fxena.treehouse.gi.ucsc.edu%3A443].

## Abbreviations

BCR: B cell receptor
CC: Cervical cancer
ccRCC: Cell renal cell carcinoma
CIN: Cervical intraepithelial neoplasia
CSCC: Cervical squamous cell carcinoma
CXCL2: Chemokine (C-X-C motif) ligand 2
DEIRGs: Differentially expressed immune-related genes
FCRL1: Fc receptor-like 1
GO: Gene ontology
KEGG: Kyoto encyclopedia of genes and genomes
LUCAT1: Lung cancer-associated transcript 1
mo-MDSCs: Monocytic MDSCs
OS: Overall survival
PCC: Pearson correlation coefficient
PS: Prognostic score
TCGA: The cancer genome Atlas
TCR: T cell receptor
